# A new teleosaurid (Crocodylomorpha, Thalattosuchia) from the Sibumasu Terrane of Southeast Asia and a taxonomic reassessment of *Indosinosuchus*

**DOI:** 10.7717/peerj.20944

**Published:** 2026-03-20

**Authors:** Komsorn Lauprasert, Apirut Nilpanapan, Jeremy E. Martin, Julien Claude, Kamonlak Wongko, Kantanat Trakunweerayut, Nuntida Dobutr, Sita Manitkoon, Supanut Bhuttarach, Thanit Nonsrirach

**Affiliations:** 1 Department of Biology, Faculty of Science, Mahasarakham University, Maha Sarakham, Thailand; 2 Excellence Center in Evolution of Life, Basin Studies and Applied Palaeontology, Mahasarakham University, Maha Sarakham, Thailand; 3 Université Claude Bernard Lyon1, LGL-TPE, UMR 5276, CNRS, ENSL, UJM, Villeurbanne, France; 4 Institut des Sciences de l’Évolution de Montpellier, Université de Montpellier, CNRS, IRD, Montpellier, France; 5 Mineral Resources Office Region 1, Department of Mineral Resources, Lampang, Thailand; 6 Palaeontological Research and Education Centre, Mahasarakham University, Maha Sarakham, Thailand

**Keywords:** *Indosinosuchus*, Khlong Min Formation, Sibumasu, Phu Kradung Formation, Indochina

## Abstract

We describe teleosaurid remains from the Middle to Upper Jurassic Khlong Min Formation at Ban Nam Pun in southern Thailand and define *Indosinosuchus peninsularensis* sp. nov. This new species is diagnosed by a unique combination of cranial and postcranial features, including the nasal reaching anteriorly at the level of the 18th maxillary alveolus, absence of an incisive foramen, oval-shaped external nares, and broad ornamented osteoderms. Phylogenetic analysis positions *I. peninsularensis* within a polytomy alongside *Mystriosaurus laurillardi* and other Asian teleosaurids, supporting a monophyletic Teleosauroidea. In addition, the revision of *Indosinosuchus kalasinensis* as a junior synonym of *I. potamosiamensis* also strengthens the taxonomic framework of the genus *Indosinosuchus*. *Indosinosuchus peninsularensis* sp. nov., discovered in a low-energy lagoonal environment, offers significant insights on the ecological preferences of teleosaurids. In contrast to the fluvially deposited Phu Kradung Formation of northeastern Thailand, the lagoonal and marginal marine sediments of the Khlong Min Formation are considered indicative of a broad spectrum of habitat occupation by Asian teleosauroids. Based on these observations, a high degree of ecological plasticity has been inferred for Thai teleosaurids, and their extensive dispersal across both the Sibumasu and Indochina terranes during the Middle to Late Jurassic has been further substantiated. The recognition of *I. peninsularensis* sp. nov. contributes to our understanding of teleosaurid diversity and paleobiogeography in Southeast Asia.

## Introduction

The Thalattosuchia, longirostrine marine crocodylomorphs, are one of the most intriguing groups that evolved during the Mesozoic (*e.g.*, Eudes-Deslongchamps, 1870; Andrews, 1909; Westphal, 1962; Buffetaut, 1982; Gasparini, Vignaud & Chong, 2000; Mueller-Töwe, 2005; Mueller-Töwe, 2006; Pierce & Benton, 2006). The thalattosuchians are mostly marine crocodylomorphs from the Early Jurassic to Early Cretaceous, present on nearly all continents. They are a unique group of crocodylomorphs highly adapted to the marine environment (*e.g.*, Von Sömmerring, 1814; Cuvier, 1824; Geoffroy Saint-Hilaire, 1831; Wilberg, 2015; Young, Sachs & Abel, 2018; Young et al., 2024a; Young et al., 2024b; Martin et al., 2019; Sachs et al., 2019; Johnson, Young & Brusatte, 2020; Johnson et al., 2022). In addition, the two major groups within Thalattosuchia, *i.e.,* Teleosauroidea and Metriorhynchoidea, possess differing levels of adaptation to the marine environment (Andrews, 1909; Andrews, 1913; Buffetaut, 1982; Hua, 1999; Young & de Andrade, 2009; Young et al., 2010; Wilberg, 2015; Brusatte et al., 2016; Fanti et al., 2016; Martin et al., 2016; Ősi et al., 2018; Sachs et al., 2019; Johnson, Young & Brusatte, 2020; Séon et al., 2020; Young et al., 2024a; Young et al., 2024b). Teleosauroids were a successful group of mostly semiaquatic thalattosuchians (Andrews, 1909; Andrews, 1913; Buffetaut, Termier & Termier, 1981; Lepage et al., 2008; Young et al., 2016; Jouve et al., 2016; Johnson et al., 2018; Foffa et al., 2019; Séon et al., 2020; Johnson, Young & Brusatte, 2020; Johnson et al., 2022; Young et al., 2024a; Young et al., 2024b). They exhibit limited skeletal adaptations for a marine lifestyle and are primarily found in estuarine and coastal deposits (Wilberg, 2015; Martin et al., 2016; Ősi et al., 2018; Foffa et al., 2019; Sachs et al., 2019; Johnson, Young & Brusatte, 2020; Séon et al., 2020; Young et al., 2024a; Young et al., 2024b). Metriorhynchoids, especially the metriorhynchids, possess the most extensive marine adaptations of any archosaurian lineage, including key features like their shark-like tail fluke (Le Mort et al., 2025) for propulsion, paddle-like limbs coupled with the loss of osteoderms for hydrodynamic efficiency, and the presence of enlarged salt glands for osmoregulation (Langston, 1973; Wilberg, 2015; Ősi et al., 2018; Sachs et al., 2019; Sachs et al., 2021; Séon et al., 2020). Fossil remains of teleosauroids are mostly known from lagoons and coastal marine environments, but they have also been found in estuarine and freshwater ecosystems, with their fossil record indicating a worldwide distribution across Africa, Asia, and Europe (Westphal, 1962; Young, 1948; Li, 1993; Buffetaut et al., 1994; Storrs & Efimov, 2000; Young, Sachs & Abel, 2018; Martin et al., 2019; Johnson, Young & Brusatte, 2020).

Historically, the first reported Asian teleosauroid was discovered from the Lower Jurassic (Toarcian) Ziliujing Formation of Szechuan, China (Li, 1993). Previously, in Thailand, a slender curved femur was reported by Buffetaut and his colleagues in 1994 from the Middle Jurassic Khlong Min Formation in the southern Peninsula. This material has previously been mentioned as closely resembling a femur of a teleosaurid (Buffetaut et al., 1994; Cuny et al., 2009) but has not been studied in detail yet. A decade later in 2004, fossil remains of a longirostrine thalattosuchian, *i.e.,* an anterior part of the upper jaw (PRC 205) and a limestone block of vertebrae and osteoderms (PRC 206), were discovered from the Ban Nam Pun site, Bang Khan District, Nakhon Si Thammarat Province (Sibumasu Terrain). All of these materials are housed at the Palaeontological Research and Education Centre (PRC), Mahasarakham University, northeastern Thailand. Nevertheless, Thai teleosaurids from the Sibumasu Terrane have not yet been described. After that, in 2008, more teleosaurid materials from the Upper Jurassic–Lower Cretaceous Phu Kradung Formation were found at the Phu Noi locality, northeastern Thailand, where they surprisingly revealed to have inhabited premanently in a freshwater habitat (Martin et al., 2016). Ten years from their initial discovery, these specimens were recognized as the first authentic Thai teleosaurid in the genus *Indosinosuchus* (Martin et al., 2019). Until now, two taxa of *Indosinosuchus* were recognized at a single locality, that is, *I*. *potamosiamensis* and *I*. *kalasinensis* (Martin et al., 2019; Johnson, Young & Brusatte, 2020).

To understand the paleobiogeographic pattern and the diversity of the teleosaurids in Thailand and Southeast Asia during the Jurassic, here we prepared and described teleosaurid material discovered in 2004 from the Sibumasu Terrane at the Ban Nam Pun site (PRC 205 and 206), Bang Khan District, Nakhon Si Thammarat Province.

## Geological Setting

**Figure 1:**
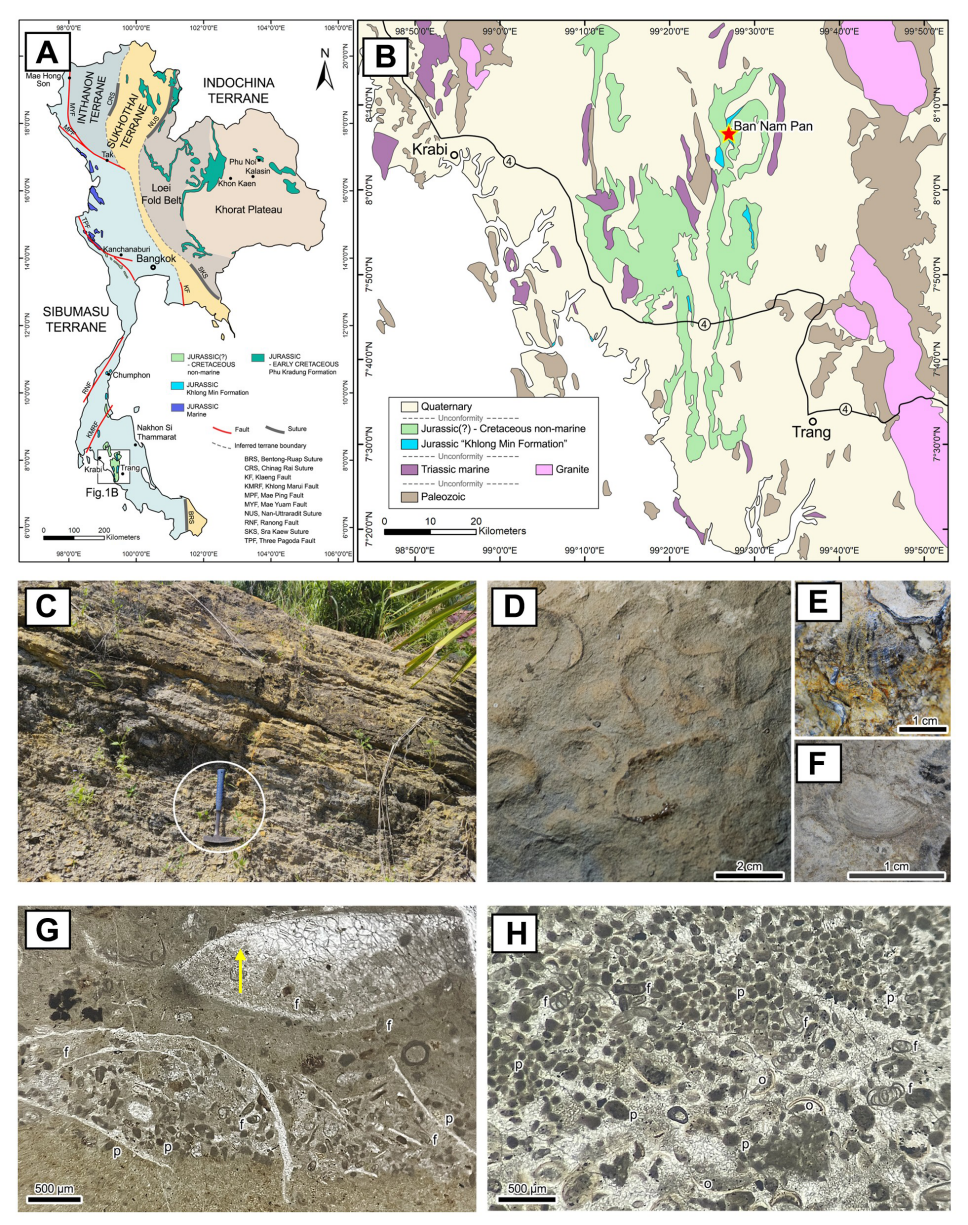
Locality, lithostratigraphy, and petrographic characteristics of the fossiliferous limestone within the Khlong Min Formation at Ban Nam Pun, southern Thailand. (A) Tectonic subdivision map of Thailand (modified after Udchachon et al., 2022), illustrating the distribution of Jurassic sedimentary rock units (based on Meesook & Saengsrichan, 2011). The Khlong Min Formation, including the Ban Nam Pun locality, is situated within the Sibumasu Terrane, whereas the Phu Kradung Formation and its equivalents are located within the Indochina and Sukhothai Terranes. (B) Simplified geological map of the Krabi–Khlong Thom–Trang region (adapted from Thailand Department of Mineral Resources, 1999; Meesook & Saengsrichan, 2011), with the study site marked by a red star. (C) Field photograph of the fossiliferous limestone outcrop intercalated with mudstone. The rock hammer (circled) is 30 cm in length for scale. (D–F) Close-up view of fossiliferous limestone rich in bivalves, (D) *Modiolus* sp. assemblage, (E) *Actinostreon* sp. (F) *Protocardia* sp. (G) MF1: Bioclast wackestone characterized by shell fragments, benthic foraminifera (f), and peloids (p). The yellow arrow indicates a bivalve shell with geopetal fabric: the lower portion infilled with micritic matrix and peloids, and the upper portion filled with sparry calcite cement. (H) MF2: Bioclast-peloidal packstone to grainstone, dominated by peloids (p) and benthic foraminifers (f) with minor ostracod shells (o).

Thailand is situated at the junction of two major continental blocks: the Sibumasu Terrane to the west and the Indochina Terrane to the east, which are separated by the Inthanon Zone and the Sukhothai Fold Belt (Fig. 1A). Both terranes represent fragments originally derived from the northern margin of Gondwana (Sone & Metcalfe, 2008; Gardiner et al., 2016). The Sibumasu Terrane was rifted away during the Early–Middle Permian and subsequently drifted northward, carrying a characteristic assemblage of warm-water faunas and shallow-marine carbonates. In contrast, the Indochina Terrane preserves a complex record of Paleozoic to Mesozoic tectonism, including extensive volcanic and plutonic activity linked to the evolution of the Sukhothai Arc (Chonglakmani, 2011; Meesook & Saengsrichan, 2011; Burrett, Udchachon & Thassanapak, 2021).

During the Mid–Late Paleozoic, these continental fragments became progressively isolated from peri-Gondwana, and their eventual collision in the Late Triassic produced the Indosinian Orogeny (Sone & Metcalfe, 2008; Gardiner et al., 2016; Burrett, Udchachon & Thassanapak, 2021). This tectonic event was intimately related to the closure of the Paleo-Tethys Ocean across Southeast Asia. One of its most significant consequences was the termination of marine deposition in the Indochina Terrane by the end of the Triassic, whereas widespread shallow-marine environments persisted on the Sibumasu Terrane well into the Middle Jurassic (Chonglakmani, 2011; Meesook & Saengsrichan, 2011). Subsequent tectonic reorganization established the present configuration of terranes across mainland Southeast Asia, with the Inthanon Zone marking a major suture and the Sukhothai Fold Belt representing remnants of a Paleo-Tethyan volcanic arc system (Metcalfe, 2013; Burrett, Udchachon & Thassanapak, 2021). These features provide crucial evidence for understanding the paleogeographic evolution of the region and form the broader geological framework in which the studied formations are situated.

**Figure 2:**
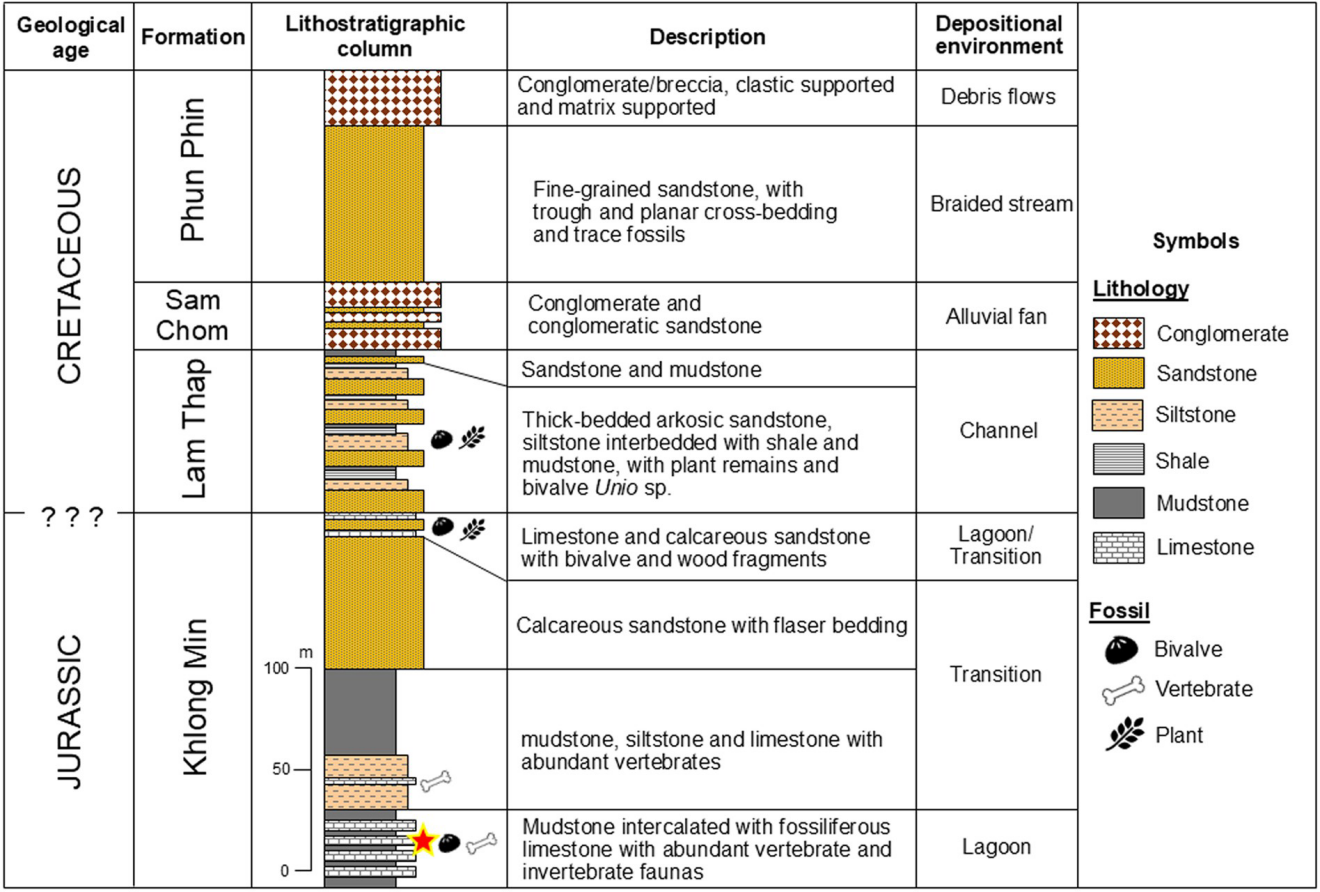
Stratigraphy of the Mesozoic Thung Yai Gorup, southern Thailand (modified after Teerarungsigul, 1999). The newly discovered teleosaurid is marked with a star symbol.

Raksaskulwong (2002) established the name Thung Yai Group for the Mesozoic succession in southern Thailand. Previously, Raksaskulwong, Teerarungsigul & Thanphisit (1989) and Teerarungsigul (1999) investigated the stratigraphy of the Mesozoic sediments in the Nakhon Si Thammarat–Krabi area (Fig. 2). The lowermost unit of the Thung Yai Group is the Khlong Min Formation, which consists of mudstone intercalated with fossiliferous limestone. Based on the lithostratigraphy and bivalve fossil assemblages, indeterminate oysters (*Juranomia* sp.), ostracods (*Darwinulla* sp.), palynomorphs, and vertebrate faunas, the Khlong Min Formation was thought to have been a lagoonal to lacustrine environment during the Middle to Late Jurassic (Meesook & Saengsrichan, 2011). The new teleosaurid remains described here come from the Khlong Min Formation near Ban Nam Pun, Bang Khan District, Nakhon Si Thammarat Province (Fig. 1B). The thin-bedded fossiliferous limestone (Fig. 1C) contains both vertebrate fossils and the *Modiolus* bivalve assemblage (Figs. 1D–1F).

### Age of Ban Nam Pun locality, Khlong Min Formation

The age of the Khlong Min Formation was long considered as Middle to Upper Jurassic. The discovery of an elasmobranch shark fauna from the lower part of the Khlong Min Formation near Ao Min village, 20 km north of Nam Pun village, supports a Bathonian–Callovian age (late Middle Jurassic) (Cuny et al., 2009; Cuny et al., 2014). According to lithological features, the fossiliferous limestone intercalated with mudstone, the Ban Nam Pun locality can be correlated to the lower part of Khlong Min Formation (Raksaskulwong, Teerarungsigul & Thanphisit, 1989; Teerarungsigul, 1999) and a late-Middle to early–Late Jurassic age is likely.

## Materials and Methods

**Figure 3:**
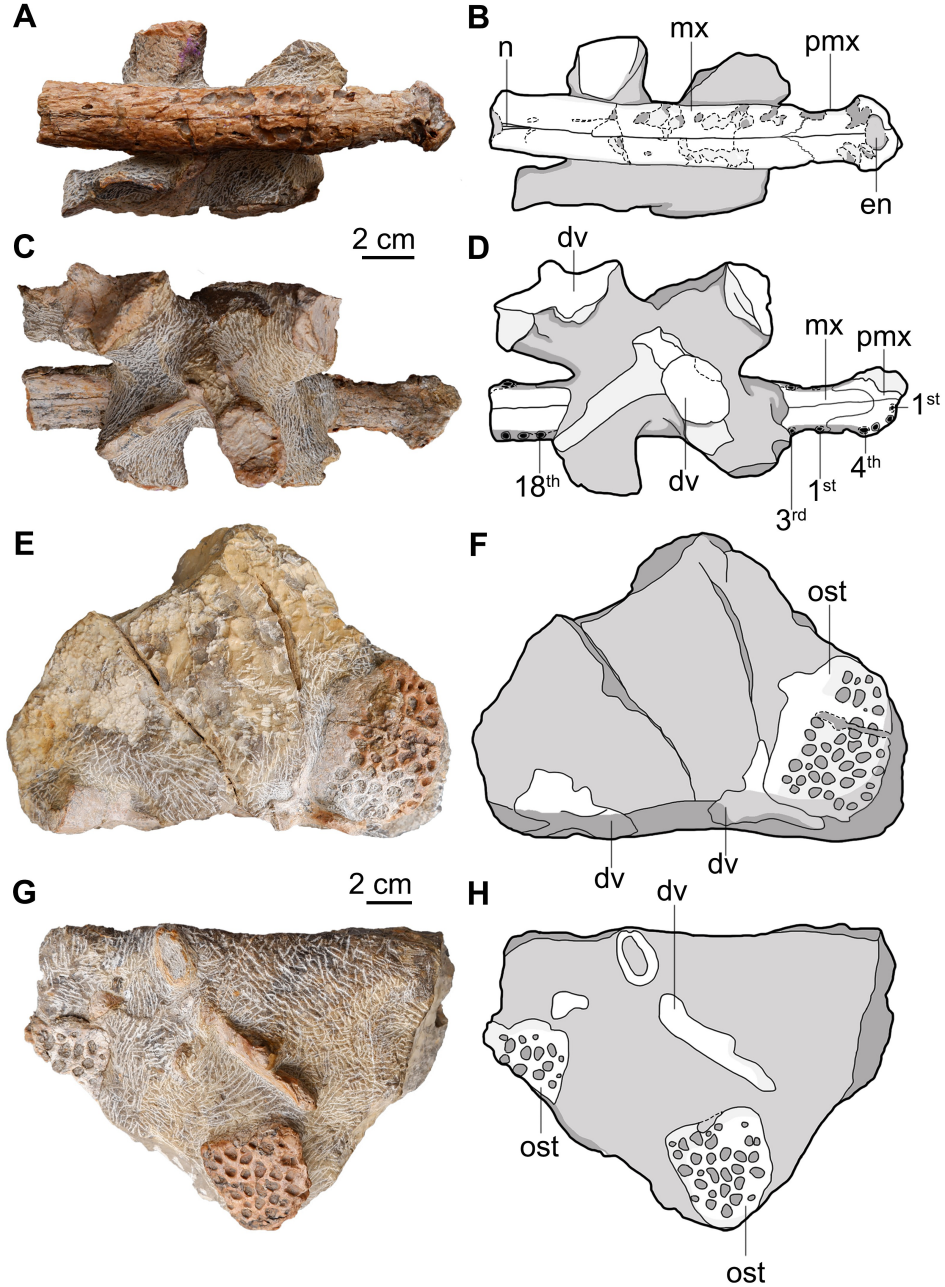
*Indosinosuchus peninsularensis* sp. nov. from the Khong Min Formation, Ban Nam Pun, southern Thailand. Photographs and interpretive drawings of *Indosinosuchus peninsularensis* sp. nov. from the Khong Min Formation, Ban Nam Pun, Nakhon Si Thammarat Province, southern Thailand. PRC-205: anterior rostrum in dorsal view (A, B) and dorsal vertebrae in ventral view (C, D). PRC-206: osteoderms and dorsal vertebrae in dorsal view (E, F) and ventral view (G, H). Abbreviations: dv, dorsal vertebrae; en, external nares; mx, maxilla; n, nasal; ost, osteoderm; pmx, premaxilla.

**Figure 4:**
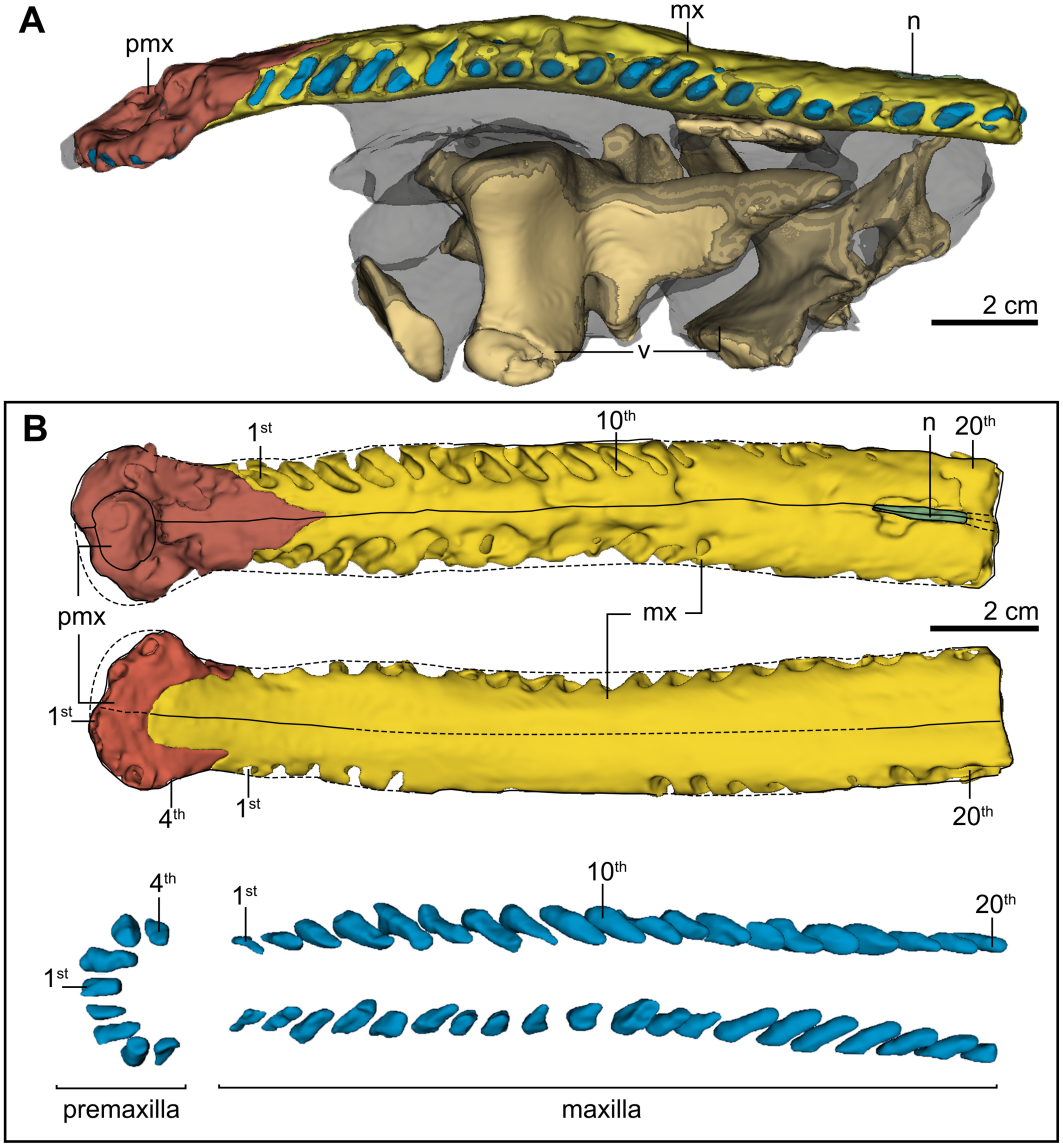
Three-dimensional cranial morphology of PRC-205. Three-dimensional anterior rostrum morphology of the holotype *Indosinosuchus peninsularensis* sp. nov. (PRC-205) from the Khlong Min Formation, Ban Nam Pun, Nakhon Si Thammarat Province, southeastern Thailand: (A) lateral view of the anterior rostrum and associated vertebrae embedded in matrix; (B) dorsal (upper) and ventral (middle) views showing the premaxilla (pmx), maxilla (mx), and nasal (n) while the lowermost shows dorsal views of premaxillary and maxillary alveolar reconstructions with surrounding matrix.

**Figure 5:**
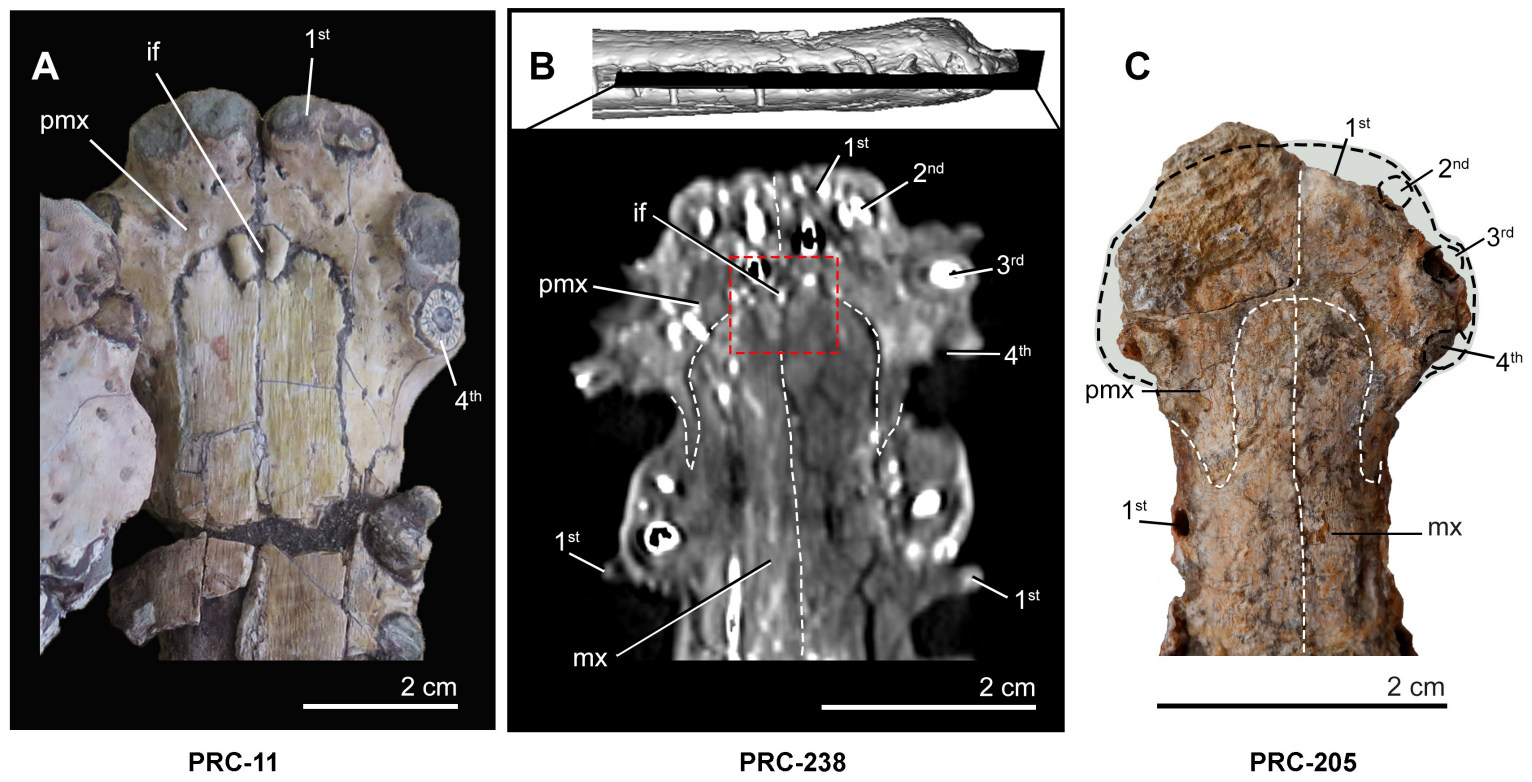
Incisive foramen comparison in *Indosinosuchus*. Comparison of the incisive foramen in *Indosinosuchus*: (A–B) Late Jurassic–Early Cretaceous Phu Kradung Formation, Phu Noi locality, Kalasin Province—(A) PRC-11 (holotype; Martin et al., 2019); (B) CT transverse view showing the incisive foramen region (dashed red box) and the premaxilla–maxilla suture in PRC-238, a specimen comparable in size to PRC-205. (C) Middle Jurassic Khlong Min Formation, Nam Pun site, Nakhon Si Thammarat Province—absence of the incisive foramen in PRC-205. Abbreviations: if, incisive foramen; pmx, premaxilla; mx, maxilla.

PRC 205, a limestone block preserving an anterior rostrum and dorsal vertebrae (Figs. 3A–3D, 4), and PRC 206, a block preserving osteoderms and dorsal vertebrae (Figs. 3E–3H, 5), originate from Ban Nam Pun, Nakhon Si Thammarat Province, southern Thailand. These specimens were found in 2004 by the Thai-French Palaeontological expedition team. A slight lateral curvature of the maxilla in PRC-205 may reflect post-depositional distortion; however, its consistent thickness throughout suggests the original shape and proportions are largely preserved. Several subcircular marks on the dorsal surface of the partial rostrum result from the erosion of the alveolar roof. The specimens were extracted from the matrix, prepared by a pneumatic air pen, and then immersed in a 10% acetic acid solution. All specimens were housed in the collection of the Palaeontological Research and Education Centre at Mahasarakham University.

The electronic version of this article in Portable Document Format (PDF) will represent a published work according to the International Commission on Zoological Nomenclature (ICZN), and hence the new names contained in the electronic version are effectively published under that Code from the electronic edition alone. This published work and the nomenclatural acts it contains have been registered in ZooBank, the online registration system for the ICZN. The ZooBank LSIDs (Life Science Identifiers) can be resolved and the associated information viewed through any standard web browser by appending the LSID to the prefix http://zoobank.org/. The LSID for this publication is: urn:lsid:zoobank.org:pub:03D87444-73F4-457F-87FF-170B4E92187A. The online version of this work is archived and available from the following digital repositories: PeerJ, PubMed Central SCIE and CLOCKSS.

### Computed tomography and digital segmentation

A tomographic analysis was performed for PRC 205 and 206 in February 2024 at the Suddhavej Hospital, Faculty of Medicine, Mahasarakham University. A computed tomography (CT) scan of the teleosaurid material was performed using a Canon Prime SP Aquilion scanner, operating at an acceleration voltage of 120 kilovolts peak (kVp) and 250 milliamperes (mA), with a slice thickness of 0.5 mm. The resulting data set for PRC-205 comprises dimensions of *x* = 132, *y* = 132, and *z* = 192, while that for PRC-206 measures *x* = 180, *y* = 180, and *z* = 214. The data output (in DICOM format) were reconstructed using the 3D Slicer software 5.6.2 (version 32448). Distinct bone features were identified and digitally segmented using the ‘Segment Editor’ module in 3D Slicer, employing thresholding and paint tools to enhance visualisation. A three-dimensional model of the skull fragment was subsequently reconstructed. Detailed information on this software version is available on the official 3D Slicer website: https://download.slicer.org/. Data were reconstructed using 3D Slicer software. The data are archived with the authors at Mahasarakham University (data archiving statement).

### Reassessment of taxonomic status

Specimen PRC-239, originally assigned to *Indosinosuchus potamosiamensis* by Martin et al. (2019), then assigned to *Indosinosuchus kalasinensis* by Johnson, Young & Brusatte (2020), was fully prepared in 2025 by removing it from its partially opened plaster jacket at the Palaeontological Research and Education Centre, Mahasarakham University. A detailed morphological reassessment was then conducted using direct comparisons with the holotype (PRC-11) and other referred specimens of *Indosinosuchus potamosiamensis*. Particular attention was given to the rostral outline, orbital morphology, neurovascular foramina, and the external mandibular fenestra. All measurements were taken with digital calipers to the nearest 0.1 mm. Taphonomic distortion was evaluated through side-by-side comparisons of symmetrical features, and character states were reassessed to determine whether previously identified differences represented true taxonomic variation or post-mortem deformation.

### Phylogenetic method

To evaluate the phylogenetic position of *Indosinosuchus peninsularensis* sp. nov., this new species was incorporated into the morphological data matrix originally compiled by Johnson, Young & Brusatte (2020), which comprises 153 crocodylomorph taxa scored for 502 morphological characters. The outgroup taxon used in the analysis was *Eopneumatosuchus colberti* Crompton & Smith, 1980. All characters were treated as unordered and unweighted to minimize bias about character evolution. The complete set of character coding for *I*. *peninsularensis* is provided in Supplemental Information.

As part of a revised taxonomic framework, *Indosinosuchus kalasinensis* (Johnson, Young & Brusatte, 2020), was excluded from the matrix, as it is now considered a junior synonym of *Indosinosuchus potamosiamensis* (Martin et al., 2019). Further discussion of this synonymy is presented in the section below. Phylogenetic analyses were carried out using TNT version 1.6 (Goloboff & Morales, 2023), following the methodology of Johnson, Young & Brusatte (2020). A heuristic search was performed utilizing 1,000 repetitions of Wagner tree construction, followed by tree bisection-reconnection (TBR) branch-swapping. The most parsimonious trees (MPTs) derived from the initial search were retained in memory and underwent a subsequent round of TBR branch-swapping to guarantee comprehensive exploration of tree space.

### Petrographic analysis

Petrographic thin sections were produced from the limestone rock that surrounds these elements in order to better understand their depositional context. Rock samples were cut into rock slabs with a diamond saw. One surface of each slab was sequentially ground flat using 200, 400, and 600 grit silicon carbide powders in ascending order. The prepared rock slab was then mounted onto a glass slide with epoxy resin. Finally, the sample was polished after its thickness was reduced to about typically 0.03 mm. The petrographical texture was interpreted and carried out under a polarizing microscope at the Laboratory in the Palaeontological Research and Education Centre, Mahasarakham University. The rocks are classified according to the classification of Dunham (1962), and the depositional environment is interpreted according to the criteria of Flugel (2010).

### Provenance and age microfacies and paleoenvironment

To better understand the stratigraphic age and palaeoenvironmental conditions of the teleosaurid-bearing unit at Ban Nam Pun, Bang Khan District, two thin-section microfacies samples were examined. Microfacies 1 and 2 yield important sedimentological and petrographic data that contribute to constraining both the depositional environment of the fossil-bearing strata (Fig. 2), as detailed below.

#### Microfacies 1 (MF1): bioclast wackestone (Fig. 1G)

This microfacies was identified from the sample PRC-206, which the new teleosaurid remains described herein come from. The MF1 is characterized by micrite matrix-supported with allochems varying from 20% to 30%. The main allochems are shell fragments and small benthic foraminifera milliolids. Peloid is a subordinate non-skeletal allochem. The micrite matrix-supported texture and small benthic foraminifera and shell fragments suggest accumulation in low-energy lagoonal settings (Flugel, 2010).

#### **Microfacies 2(MF2):Bioclast-Peloidal Packstone to Grainstone** (Fig. 1H)

A rock sample close to the locus that yielded the fossil crocodile, collected in 2024, was processed into a thin section for microfacies analysis. The MF2 is characterized by 15–20 cm of thin-bedded limestone, which is brownish to light grey in colour (Fig. 1C). Under the microscope, MF2 shows grain supported packstone texture and lacks micrite and grainstone. The allochems range between 40% to 60% and consist of abundant peloid and benthic foraminifera milliolids. The packstone–grainstone texture indicates deposition in shallow water depth above a fair-weather wave base, with medium to high energy conditions (Flugel, 2010). The abundance of peloids associated with benthic foraminifera indicate peritidal settings (Martini et al., 2007).

### Systematic Palaeontology

**Table:** 

**Figure 6:**
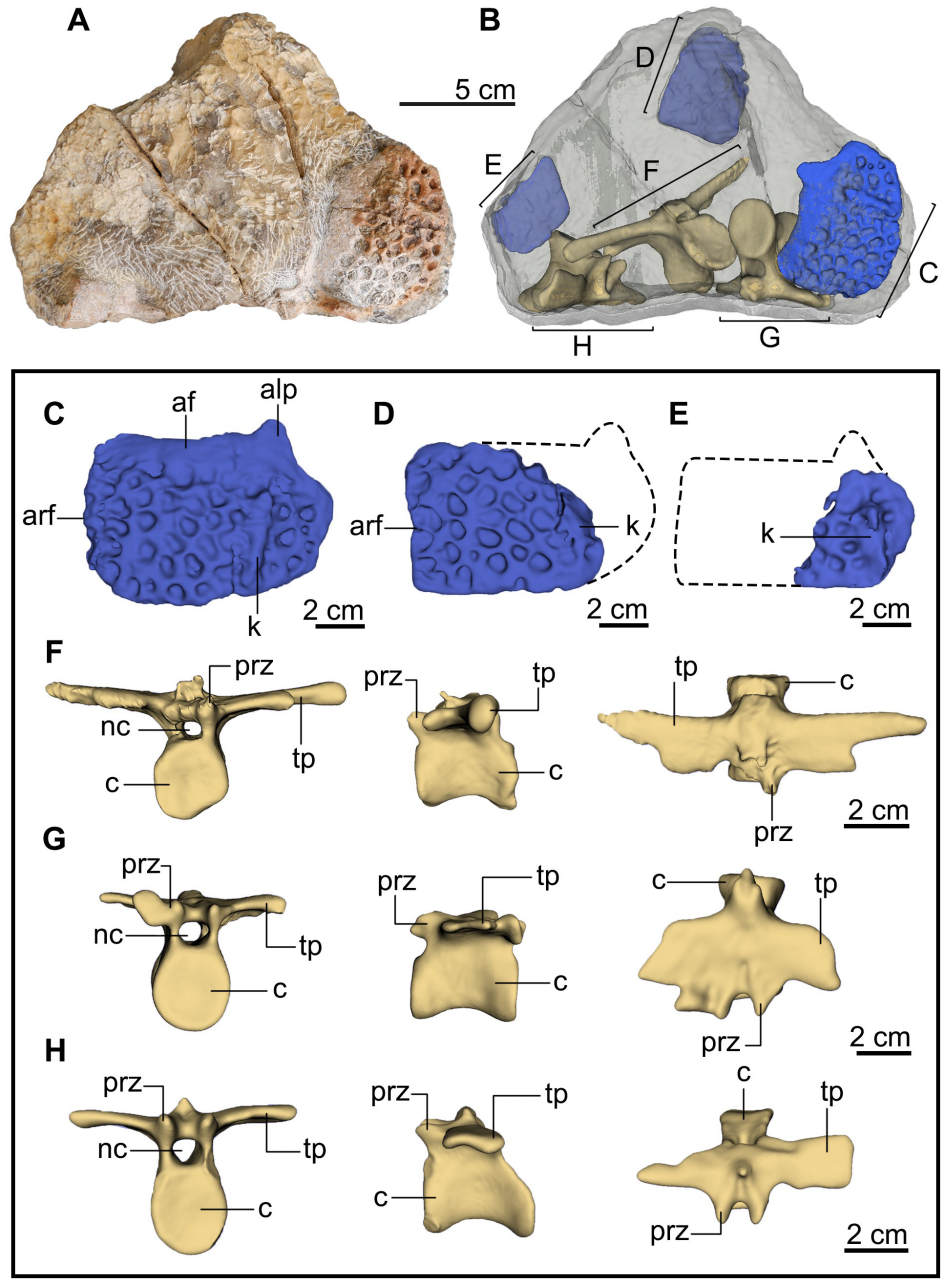
Holotype vertebrae and osteoderms of *Indosinosuchus peninsularensis*. Photograph (A) and reconstructions based on computed tomography (CT) data (B) of the holotype specimen (PRC-206) of *Indosinosuchus peninsularensis* sp. nov. in dorsal view, showing the vertebrae and associated osteoderms embedded in matrix. Isolated osteoderm elements are shown in (C–E) while individual dorsal vertebrae (F–G) are illustrated in anterior, right lateral, and dorsal views, respectively. Abbreviations: af, anterior facet; alp, anterolateral process (peg); arf, articular facet; c, centrum; k, keel; nc, neural canal; prz, prezygapophysis; tp, transverse process.

CROCODYLOMORPHA Hay, 1930
THALATTOSUCHIA Fraas, 1901
NEOTHALATTOSUCHIA Young et al., 2024a; Young et al., 2024b
TELEOSAUROIDEA Delfino & Dal Sasso, 2006
TELEOSAURIDAE Geoffroy Saint-Hilaire, 1831
Genus *Indosinosuchus*Martin et al., 2019
*Indosinosuchus peninsularensis* sp. nov. (Figs. 3–6)

**Holotype**: PRC 205 and PRC 206, comprising an anterior portion of the rostrum, osteoderms, and dorsal vertebrae.

**Type locality**: Ban Nam Pun, Bang Khan District, Nakhon Si Thammarat Province, southern Thailand.

**Stratigraphic occurrence**: Lower part of the Khlong Min Formation, Thung Yai Group, late Middle Jurassic to early Late Jurassic.

**Diagnosis**. *Indosinosuchus peninsularensis*, PRC 205, is characterized by the following combination of characters (autapomorphies denoted with*): (1) four premaxillary alveoli; (2) moderately laterally expanded premaxilla with subequal length and width, producing a broad yet unflared outline; (3) margin of premaxillary-maxillary suture is concave between the 4th premaxillary –1st maxillary alveoli; (4) the external narial opening is subcircular or slightly ‘8-shaped’ in dorsal view; (5) absence of incisive foramen at medial contact of premaxillae*; (6) anterior tip of nasals reaching the level of the 18th maxillary alveolus*.

**Etymology**—The species name “*peninsularensis* ” refers to its occurrence in the southern penisular region of Thailand.

## Description

### Skull

#### General preservation

All teleosaurid remains are preserved in a block of dense limestone (Figs. 3A–3D). The surface of the rostrum is partially eroded, but ornamentation and sutures are visible. The rostrum has been slightly deformed, as evidenced by the curvature of its anterior part, which is best seen from a lateral view. Given the medium to high energy shallow depositional setting, the cranial and postcranial elements may represent different individuals or taxa; however, the single-taxon hypothesis remains the most parsimonious interpretation. Further mechanical preparation could not be achieved without risking damage to the bone, so the specimen was computed tomography (CT) scanned (Figs. 4 and 6). The rostrum is broken off at the level of the anterior margin of the nasal. All postcranial elements are disarticulated and mixed closely with the rostrum in the same block.

#### Premaxilla

The premaxillae are nearly complete, eroding on their left anterior margin and along the posterolateral narial borders (Figs. 3A–3D and 4A–4D). Despite this preservation, they surround the external nares, as evidenced by the premaxillae contribution to the posterior margin of the narial opening. The general outline of the premaxillae is roughly oval-shaped in both dorsal and ventral views, with the elements being nearly as long as they are wide; laterally expanded margins adjacent to the narial opening give the region a complex spatulate or T-shaped appearance (Fig. 4B). Anteriorly, the premaxilla shows a distinctly subcircular narial opening that occupies most of the dorsal surface of the bone; however, a fracture along the mesial margin complicates the assessment of its original outline. Based on the preserved remains, slight traces of bulbous projections can be observed along the anteromedial and posteromedial margins, yet the available evidence is insufficient to confirm whether the narial opening truly resembled a ‘B-shape’ or ‘8-shape’ (Fig. 4). The anterolateral margin is broad, as evidenced from the right side, which is complete. The dorsal surface of the premaxilla is mostly smooth, except for the posterolateral surfaces, where shallow furrows are visible. Dorsally, the premaxillary-maxillary suture makes a wavy, ‘V’-shaped line. The alveolar count was made possible on the ventral surface thanks to the broken section along the left premaxilla (Fig. 3D) and CT reconstruction for the right premaxilla (Fig. 4). Four alveoli are visible on the left premaxilla and one on the right. A short diastema separates the first and second alveoli from the third and fourth, with interalveolar spacing differing between positions: P1–P2 are divided only by a thin lamina, whereas P3–P4 are more widely separated. The ventral premaxillary-maxillary suture is ‘U’-shaped and shows an anterior projection of the maxillae that reaches the level between alveoli four and five. The medial suture between the premaxillae and the premaxillary-maxillary suture lacks an incisive foramen (Fig. 5C). No teeth were observed, despite the alveoli being visible. The premaxillary alveoli are subequal in size, with the first and second alveoli closely spaced and separated by a thin lamina, while the third and fourth alveoli are slightly more widely spaced posteriorly. In contrast, the maxillary alveoli exhibit comparable dimensions along the preserved length of the specimen.

#### Maxilla

The maxillae preserve their anterior and mid-region, being broken off at the level of their suture with the nasals (20th maxillary alveoli). Ornamentation is subtle on the dorsal surface of the maxilla and consists of a smooth surface ornamented with a few shallow furrows. The maxillae form an almost tubular rostrum, slightly wider than tall, with straight, parallel margins extending to the nasal tip. Anteriorly, the rostrum distinctly tapers into the premaxilla (Fig. 3A), rather than exhibiting the usual forward broadening, a feature that clearly contrasts with other sympatric forms. As mentioned above, the preserved maxillary alveolar count is 20, as established from CT scan data (Fig. 4). The posterior extent of the maxillary tooth row is unknown. Therefore, the total number of maxillary alveoli was greater than 20.

#### Nasals

The nasal bones are limited to their anteriormost processes that project as a thin lamella between both maxillae. The anterior tip of the nasals reach the level of the 18th maxillary alveolus (Fig. 3B).

### Postcranial skeleton

#### Dorsal vertebrae

Three dorsal vertebrae have been recognized by the CT scan method from specimen PRC-206 (Figs. 6F–6H). The neural spine processes are impaired and can only be seen at their base. The neural canals are elliptical. All vertebrae retain well-preserved prezygapophyses; however, postzygapophyses are not preserved in any of the available specimens. The anterior surfaces of the centra are concave and higher than they are wide. The CT scan data reveal the slightly concave surfaces of the centra both anteriorly and posteriorly, confirming amphicoelous vertebrae. The centrum is relatively longer than it is high in lateral view and exhibits slightly transverse compression in dorsal view. The ventral margin of the centrum exhibits a concave shape. The transverse processes are anteroposteriorly flat and broad (Figs. 6G, 6H), and are notably elongated, particularly in the vertebra shown in Fig. 6F. The transverse process of this specimen also bears a combined capitulum and tuberculum, a feature also observed in the thoracic vertebrae of *Indosinosuchus potamosiamensis* (Martin et al., 2019; Bhuttarach et al., 2023). These morphological features strongly suggest that this vertebra is part of the thoracic series.

#### Osteoderms

In PRC-206, three osteoderms are visible (Figs. 6C–6E). The most complete specimen is large, wider than long, and subrectangular in shape (Fig. 6C). A short and flat anterolateral process (or peg) can be observed. In dorsal view, a longitudinal keel extends anteroposteriorly to the posterior edge. The anterior part of the osteoderm displays a smooth and slightly curved facet bar. Its medial margin has a straight articular facet, forming the suture between a pair of osteoderms, whereas its lateral margin is slightly convex. The pits are large, round to ovoid in outline, well separated from one another, and irregularly distributed across the dorsal surface. In each isolated osteoderm, 38 pits were counted in Fig. 6C, with an average diameter of 4.95 mm; 18 pits in Fig. 6D, averaging 5.60 mm; and 9 pits in Fig. 6E, averaging 4.02 mm. Laterally, the ornamented surface is laminar and presents a thick margin. However, the rest of the osteoderms are broken (Figs. 6D and 6E), but their morphological characteristics are similar to the complete specimen. The characteristics of embedded osteoderms in PRC-206 resemble those of the trunk osteoderms of teleosaurids, such as *Indosinosuchus* (Martin et al., 2019; Bhuttarach et al., 2023) and machimosaurids, such as *Macrospondylus bollensis* (Johnson, Young & Brusatte, 2020).

## Taxonomic Re-evaluation of *Indosinosuchus Kalasinensis*

**Figure 7:**
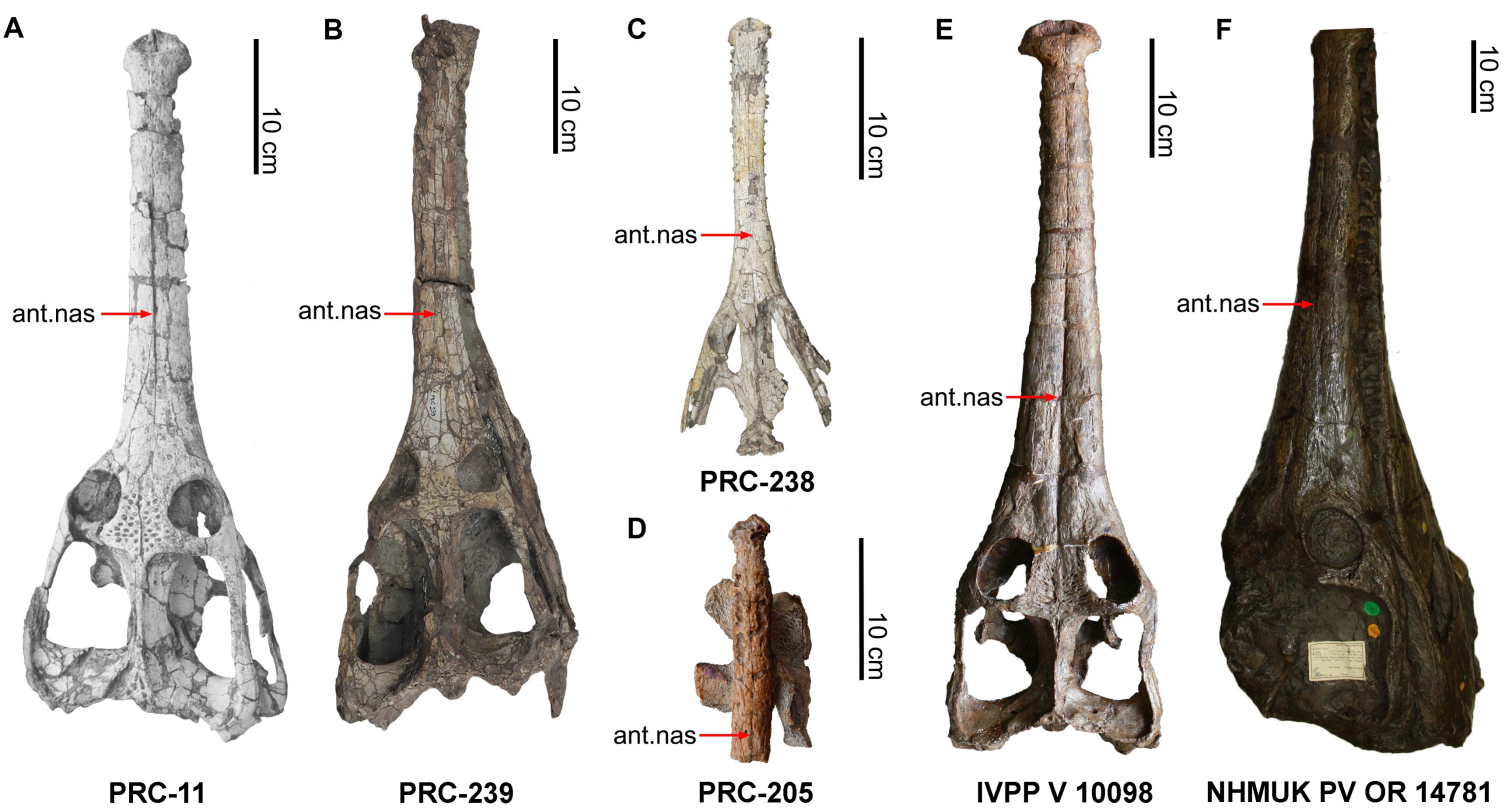
Dorsal skull comparisons of teleosaurids. Dorsal views of teleosaurid skulls. *Indosinosuchus* specimens from Thailand: (A) PRC-11 (*I*. *potamosiamensis*), (B) PRC-239 after removal from the plaster jacket, (C) PRC-238, and (D) PRC-205 (*I*. *peninsularensis* sp. nov.). Comparative specimens: (E) Chinese teleosaurid (IVPP V 10098) from the Lower Jurassic Ziliujing Formation, Sichuan, China; (F) *Mystriosaurus laurillardi* (NHMUK PV OR 14781) from the Lower Jurassic Posidonienschiefer Formation, Altdorf, Germany. Red arrows indicate the position of the anteriormost of nasal (ant. nas).

Martin et al. (2019) on the basis of all teleosaurid specimens excavated so far at the Phu Noi site, erected the taxon *Indosinosuchus potamosiamensis* as a new genus and species and designated the complete skull with a mandible (PRC 11) as the holotype (Fig. 7A). Subsequently, Johnson, Young & Brusatte (2020) conducted a phylogenetic analysis of Teleosauroidea and referred one specimen (PRC-239) from the previously recognized species as a second distinct teleosaurid species, *Indosinosuchus kalasinensis* (Fig. 7B). This taxonomic decision was based on several distinguishing characteristics, such as: the absence of immediate rostral narrowing anterior to the orbits; nearly twice-enlarged premaxillary and maxillary neurovascular foramina; ‘B’-shaped external nares in anterior view; a premaxillary length posterior to the external nares ranging from 50% to 65%; a minimum frontal width approximately equal to the orbital width; dorsal orbital margins flush with the skull dorsal surface; and a poorly elliptic external mandibular fenestra. Prior to our present study, PRC-239 was only partly prepared and available in a partially opened plaster jacket at the Palaeontological Research and Education Centre. In the present study, the specimen was fully removed from its jacket to permit a comprehensive morphological assessment and comparison with other *Indosinosuchus potamosiamensis* specimens, most notably the holotype, PRC 11.

**Figure 8:**
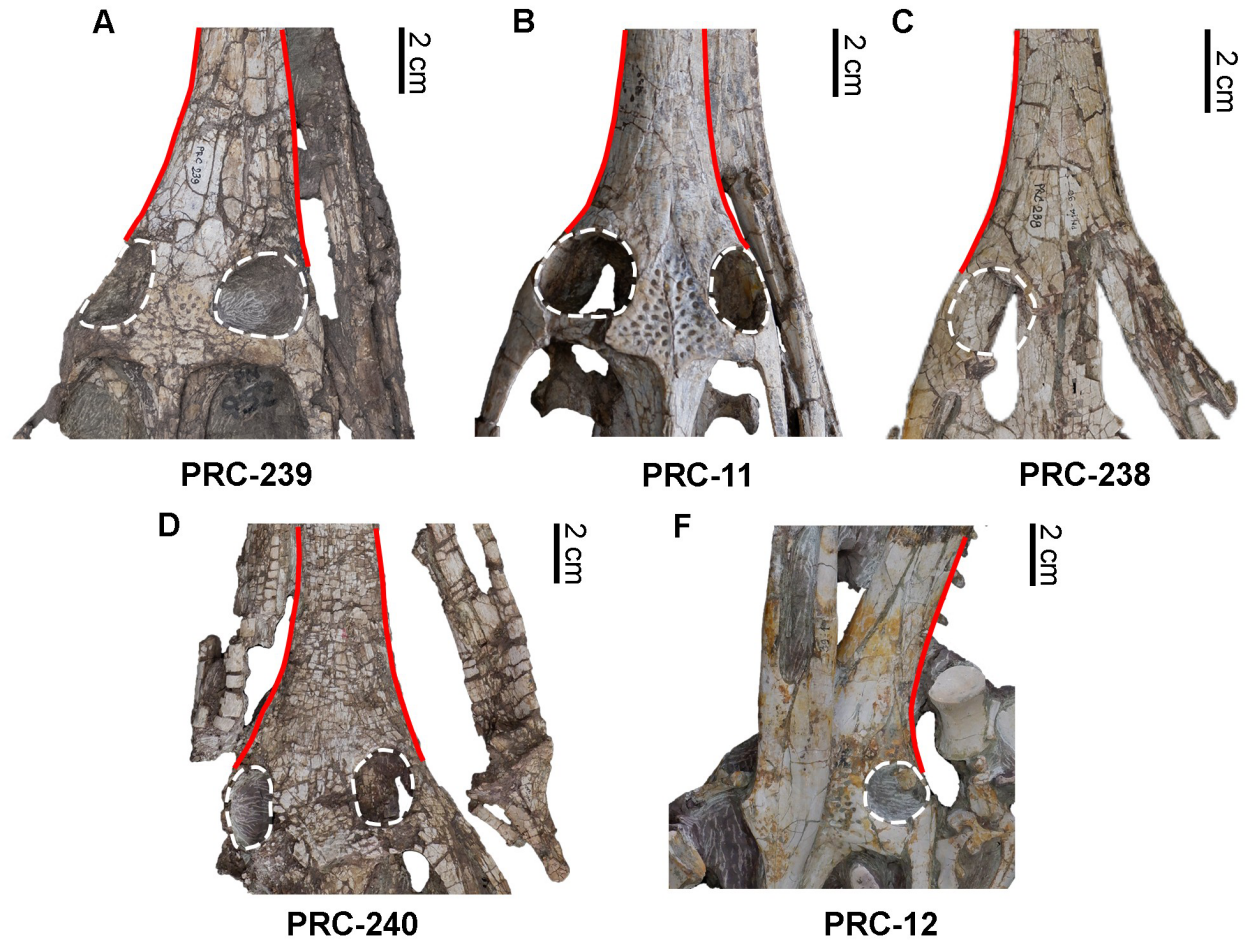
Rostrum and frontal morphology of *Indosinosuchus*. Comparison of the rostrum and frontal morphology in *Indosinosuchus*: (A) PRC 239; (B) holotype of *I. potamosiamensis* (PRC 11); (C–F) referred specimens of *I*. *potamosiamensis*: (C) PRC 238, (D) PRC 240, (F) PRC 12.

**Figure 9:**
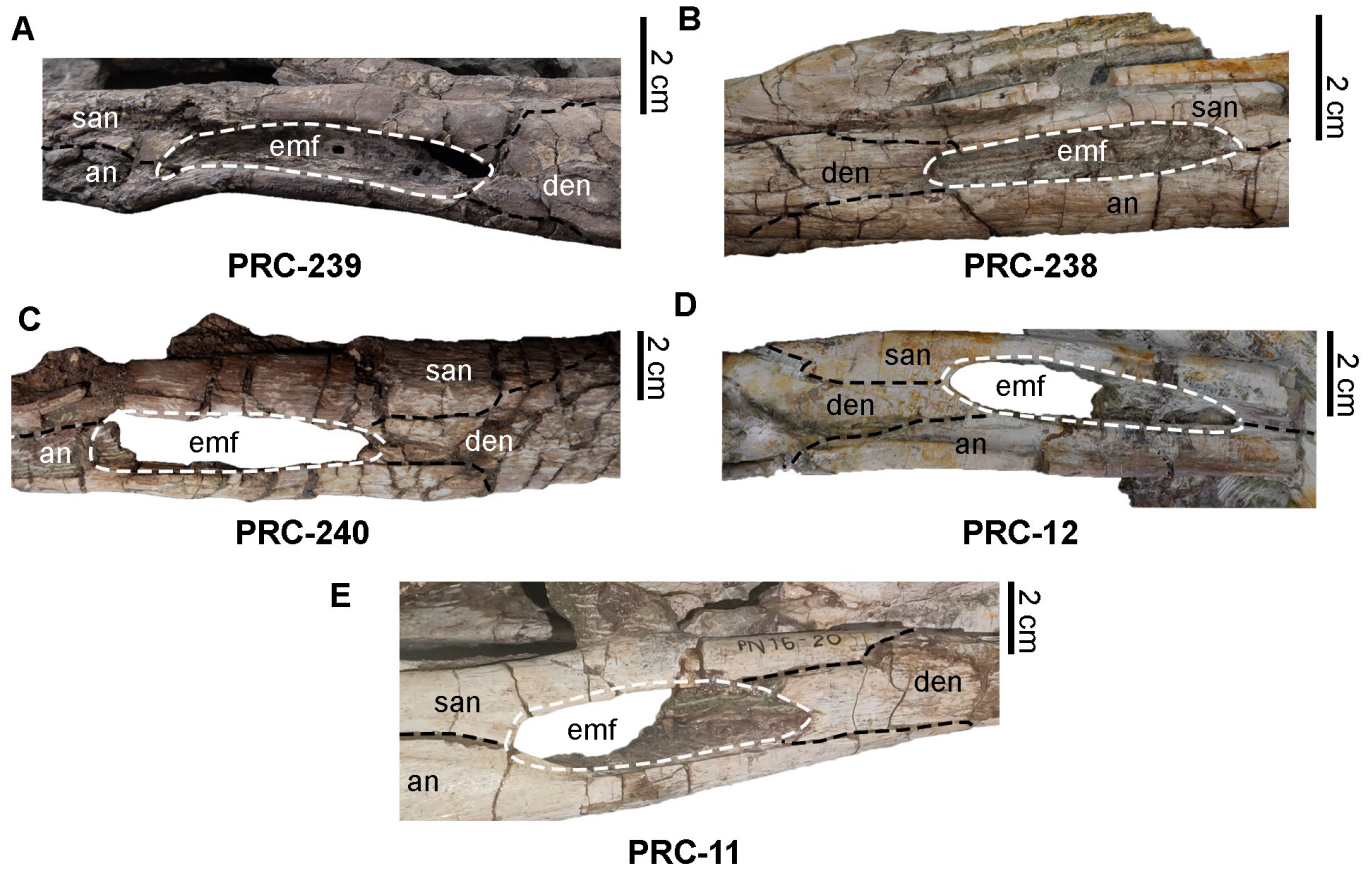
External mandibular fenestra of *Indosinosuchus kalasinensis* PRC 239 (A) and referred specimens of *I*. *potamosiamensis* (PRC 238, (B); PRC 240, (C); PRC 12, (D); holotype PRC 11, (E)), all exhibiting a uniformly elliptical morphology. Abbreviations: emf, external mandibular fenestra; san, surangular; an, angular; den, dentary.

A detailed reassessment of the morphology of PRC-239 (Fig. 8A), in comparison with the holotype (Fig. 8B) and referred specimens of *Indosinosuchus potamosiamensis* (Figs. 8C–8F), reveals that the slight constriction of the rostrum just in front of the orbits appears to be the result of taphonomic distortion rather than a true anatomical feature. In PRC-239, this constriction is absent on the right side but is notably present on the left margin. A comparable asymmetry is seen in PRC-240 (Fig. 8D), where the left margin of the rostrum shows a distinct tapering, whereas the right side remains relatively straight. These inconsistencies between the two margins in both specimens suggest that the asymmetry is attributable to post-mortem deformation during fossilization, rather than representing genuine morphological variation. The variation observed in the shape of the orbital margins (Figs. 8A–8F) and the external mandibular fenestra in PRC-239 (Fig. 9A) is best interpreted as the result of post-mortem distortion as well. As noted by Johnson, Young & Brusatte (2020), compression along the medial orbital margins is evident in this specimen. Our re-examination of all available material reveals that the frontal surface of PRC-239 (Fig. 8A) is moderately abraded and this abrasion appears to have affected adjacent cranial structures. Notably, this has likely contributed to the reduced upturning of the dorsal orbital rim in PRC-239—a condition also observed in PRC-240 (Fig. 8D)—that contrasts with the more prominently elevated rim seen in the holotype of *Indosinosuchus potamosiamensis* (Fig. 8B). Although Johnson, Young & Brusatte (2020) described the external mandibular fenestra of PRC-239 as weakly elliptical (Fig. 9A), our re-evaluation of additional specimens reveals a consistently elliptical outline across the sample set (Figs. 9B–9E). As shown in Table 1, the height and length of the fenestrae range from 0.9–2.0 cm and 5.8–12.5 cm, respectively, with PRC-239 falling within this range. The slightly larger dimensions observed in PRC-239 correspond proportionally with its overall skull size. These findings indicate that variation in fenestra shape and size is more likely a function of allometric scaling, in regard to ontogeny and taphonomic distortion, rather than diagnostic differentiation.

**Table 1:** **Comparative measurements of*Indosinosuchus*** from Phu Noi. Selected measurements (in cm) of *Indosinosuchus potamosiamensis* and specimen PRC-239. Modified from Martin et al. (2019).

**Characteristics**	**PRC**-**11**	**PRC**-**12**	**PRC**-**238**	**PRC**-**239**	**PRC**-**240**
Premaxilla length	7.24	6.76	7.33	8.90	N/A
Minimum frontal width	3.5	>2	N/A	3.4	5.6
Orbital width	4.2	3.2	3.4	4.3	4.1
Anteroposteriorly thickened postorbital bar	2.0	N/A	N/A	2.0	N/A
Premaxilla, proportion of total length posterior to the external nares	over 67%	N/A	N/A	68.8	N/A
External mandibular fenestra length	8.3	8	5.8	10.5	12.5
External mandibular fenestra height	2	1.7	0.9	1.9	1.7

**Figure 10:**
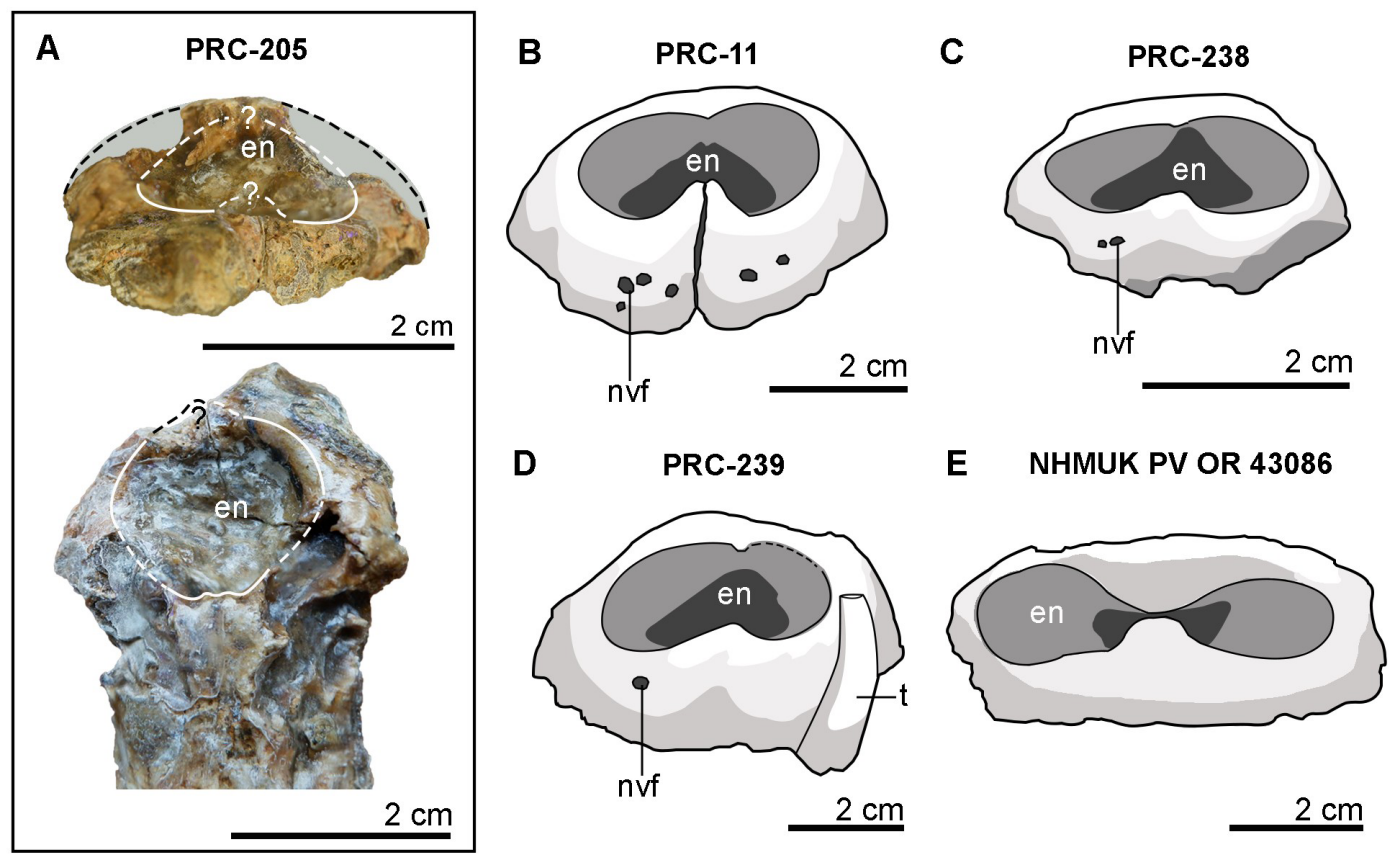
External nares morphology of *Indosinosuchus*. External nares of *Indosinosuchus peninsularensis* sp. nov. (PRC 205) in anterior (upper) and dorsal (lower) views (A). Although incomplete preservation prevents confirmation of the exact morphology, the outline appears slightly ‘8-shaped’. Comparisons are provided with the illustrations of the holotype of *I*. *potamosiamensis* (PRC 11; (B)), a referred specimen of *I*. *potamosiamensis* (PRC 238; (C)), and *Indosinosuchus kalasinensis* (PRC 239; (D)), all showing similarly slightly ‘8-shaped’ outlines. This contrasts markedly with *Bathysuchus megarhinus* (NHMUK PV OR 43086), which exhibits a strongly ‘8-shaped’ outline in anterior view. Abbreviations: en, external nares; nvf, neurovascular foramina; t, tooth.

Neurovascular foramina on the premaxilla and maxilla of *Indosinosuchus potamosiamensis* specimens (Fig. 10B) range from approximately 1–3 mm in diameter, consistent with those observed in PRC-239 (Fig. 10C). Upon closer examination, the external nares of PRC-239, as well as those of other *I*. *potamosiamensis* specimens, appear to exhibit a slightly ‘8-shaped’ outline in anterior view (Figs. 10B–10E). However, this condition is less pronounced than the distinctly ‘8-shaped’ configuration observed in aeolodontine taxa (Fig. 10F). A revised measurement of the premaxilla in PRC-239 indicates that the portion posterior to the external nares accounts for approximately 68.8% of the total premaxillary length, and not of approximately 64% as reported by Johnson, Young & Brusatte (2020). Additionally, comparisons of minimum frontal width and orbital width (Table 1) show a consistent pattern across all specimens: the frontal width is markedly narrower than the orbital width in both PRC-239 and other referred specimens of *I*. *potamosiamensis*. Lastly, the autapomorphic character of an anteroposteriorly thickened postorbital bar was re-evaluated in PRC-239 and PRC-11. The bar thickness in both specimens measures approximately two cm (Table 1). However, due to the absence or poor preservation of this feature in other specimens, its taxonomic utility remains ambiguous. As such, this character alone does not provide a reliable basis for species differentiation.

Reexamination of PRC-239 reveals a higher degree of morphological congruence with *Indosinosuchus potamosiamensis* than previously recognized. Apparent differences in the morphology of the external mandibular fenestra, orbital margins, and rostrum outline are best explained as taphonomic artifacts, which likely obscured key diagnostic features. Based on this reassessment, we propose that *Indosinosuchus kalasinensis* be regarded as a junior synonym of *Indosinosuchus potamosiamensis*.

## Phylogenetic Analysis

The original analysis by Johnson, Young & Brusatte (2020) yielded 125 MPTs of length 1,659 steps. Following the addition of *I*. *peninsularensis* and the removal of *I*. *kalasinensis*, the revised analysis produced 181 MPTs. The length of tree was reduced to 1,615 steps, indicating a decrease of 44 steps from the original result. The strict consensus tree derived from these MPTs displays a more resolved topology, with a consistency index (CI) of 0.402 and a retention index (RI) of 0.833. These values suggest a moderate degree of homoplasy while also reflecting strong character retention and a relatively good fit between the morphological data and the resulting tree structure.

**Figure 11:**
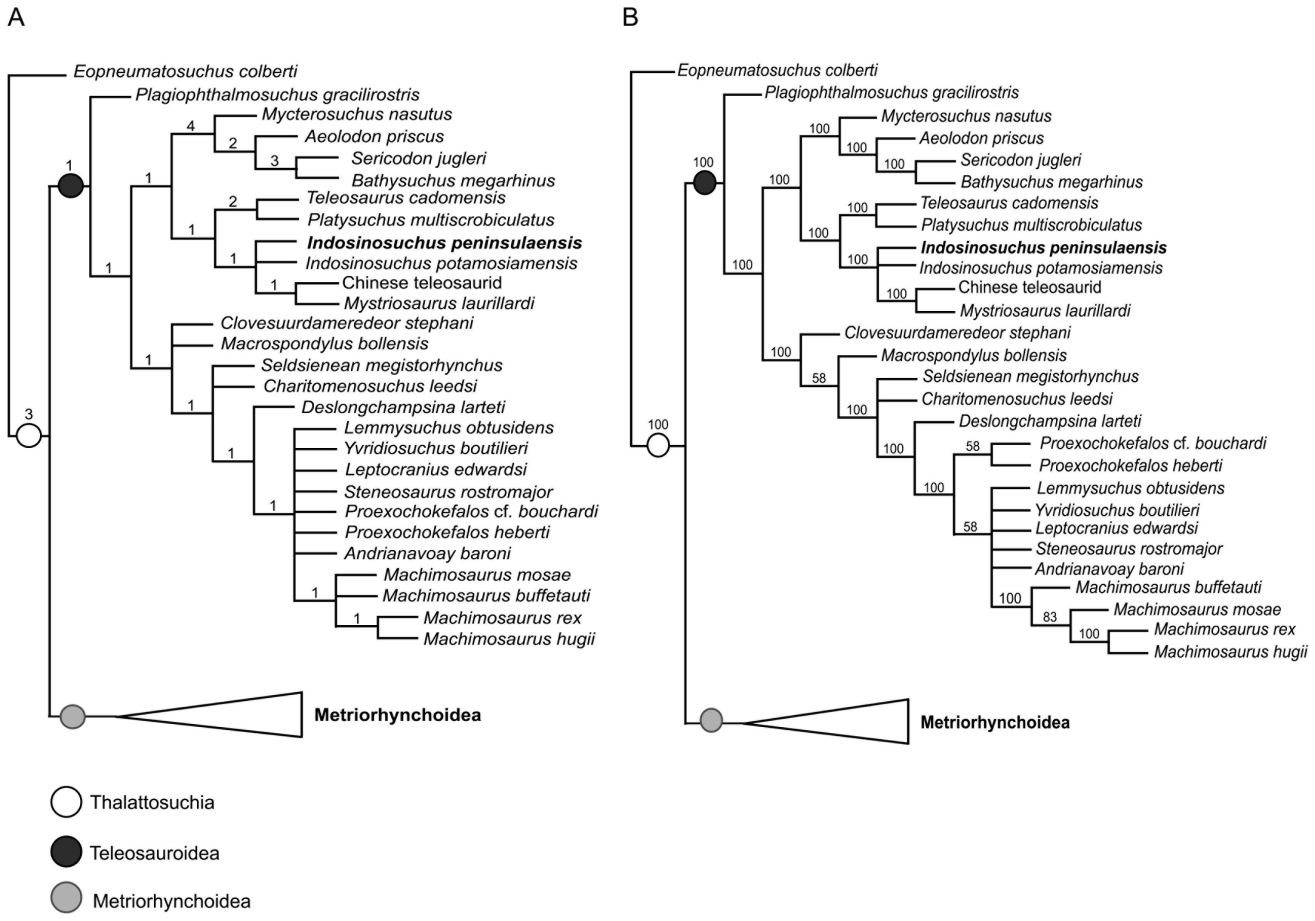
Phylogenetic relationships of *Indosinosuchus*: strict consensus and majority-rule topologies with support indices. The phylogenetic analysis, (A) simplified strict consensus topology of 181 most parsimonious to a total of 1,615 steps. Resulted in simplified strict consensus topology (consistency index (CI) = 0.402; retention index (RI) = 0.833); (B) a parsimonious majority rules topology (consistency index (CI) = 0.413; retention index (RI) = 0.840). Values in (A) denote the Bremer decay index/diagnostic characters, while values in B indicate bootstrap support percentages. Both serve as indicators of the robustness and reliability of the inferred clades.

The resulting topology of our strict consensus tree closely aligns with that proposed by Johnson, Young & Brusatte (2020), in which a monophyletic Teleosauroidea is consistently recovered near the base of Thalattosuchia (Fig. 11). Within Teleosauridae, two distinct clades—Aeolodontini (Johnson et al., 2022) and Teleosaurinae—are well resolved. The latter comprises *Teleosaurus cadomensis* and *Platysuchus multiscrobiculatus*, forming a coherent clade that shows close affinities with a polytomy including several Asian teleosaurids and *Mystriosaurus laurillardi*. One of the more intriguing aspects of our analysis is the position of a Chinese teleosaurid specimen from the Early Jurassic (IVPP V 10098), which is recovered as the sister taxon to *Mystriosaurus laurillardi*. Together, these two species form part of a polytomy alongside *Indosinosuchus peninsularensis* from the Middle to Late Jurassic and *I*. *potamosiamensis* from the Late Jurassic. While this section of the tree appears less resolved, it is supported by a Bremer support value of 1, as calculated in TNT, suggesting that the placement of these taxa is phylogenetically robust despite the appearance of polytomy.

Additional support for the close relationship among these taxa lies in several clear morphological traits based on the character list of Johnson, Young & Brusatte (2020). They show no conspicuous ornamentation, or are ornamented with an irregular pattern of ridges, rugosities, and anastomosing grooves (#11.0). In addition, the interalveolar spacing between P1–P2 and P3–P4 differs notably: P1–P2 are separated by a thin lamina, while P3–P4 are well separated (#292.1). These shared traits represent meaningful synapomorphies that reinforce their close evolutionary relationship. In addition, *Indosinosuchus peninsularensis* can be distinguished from the others by several autapomorphic features: the rostrum is notably wider than tall (the lateromedial axis exceeds the dorsoventral axis by more than 10%) (#7.0); the external nares are oval in dorsal view, with the width over 10% longer than their anteroposterior length (#35.1); and there is no incisive foramen at the medial contact of the ventral rami (#179.0).

The revised dataset, which incorporates new anatomical information from the Ban Nam Pun specimen along with updated taxonomic interpretations, provides a more parsimonious and stable hypothesis of thalattosuchian relationships. The increase in the number of most parsimonious trees likely reflects the novel combination of morphological features present in *I*. *peninsularensis* , which contributes new character variation that was not captured in previous analyses.

## Discussion

### Comparisons with other teleosaurids

At present, the family Teleosauridae consists of the Chinese teleosaurid (IVPP RV 10098) (Fig. 7E), *Indosinosuchus*, *Mystriosaurus* (Fig. 7F), and two clades, *i.e.,* Teleosaurinae and Aeolodontini (Johnson, Young & Brusatte, 2020; Johnson et al., 2022; Young et al., 2024b). Despite the anteriormost premaxilla of *Indosinosuchus peninsularensis* (PRC 205) being absent, the shape of the anterior edge between the second and third premaxillary alveoli is noticeable. The curvature is evident, with the third premaxillary alveoli positioned posterolaterally to the second premaxillary alveoli. In contrast, the members of the Aeolodontini have a sub-rectangular shape between the second and third premaxillary alveoli along their front edge. In anterior view, the premaxilla of *I*. *peninsularensis* exhibits a distinctly subcircular morphology, lacking bulbous projections along the posteromedial margins of the external nares (Fig. 10A). This condition contrasts markedly with that of aeolodontine taxa, in which the premaxilla bears two prominent bulbous projections that create a characteristic figure-eight (‘8’) configuration (Foffa et al., 2019; Johnson, Young & Brusatte, 2020). Smooth and slightly curved dorsal osteoderms found in *I*. *peninsularensis* exhibit large, round to ovoid ornamentation that is distinctly isolated from each other. These materials do not have the apomorphic features of the subfamily Teleosaurinae, which include densely arranged osteoderms with small round to ellipsoid pits and presacral dorsal osteoderms that are strongly curved (Westphal, 1961; Johnson, Young & Brusatte, 2020). As a result, *I*. *peninsularensis* is not appropriately classified within either the Teleosaurinae or Aeolodontini clades. Its distinct morphological characteristics, which deviate from those seen in both groups, suggest that it represents a separate lineage within the Teleosauridae family.

*Indosinosuchus peninsularensis* has unusual jaw characteristics that distinguish it from both European and Asian teleosaurids. Notably, the contact between the premaxilla and maxilla in *I*. *peninsularensis* is triangular (V-shaped) in dorsal view, with only slight interdigitating along the suture (Fig. 3B). This condition contrasts with the slightly interdigitating and round premaxilla-maxilla contact observed in the European teleosaurid *Mystriosaurus laurillardi* (Sachs et al., 2019) (Fig. 7F), but is shared with Asian forms, including *I*. *potamosiamensis* (Figs. 7A–7C), the Chinese teleosaurid (IVPP RV 10098) (Fig. 7E), and the holotype of *Peipehsuchus teleorhinus* (IVPP RV 48001) (Young, 1948; Plate II, Fig. 1). Furthermore, *I*. *peninsularensis* has a rostrum that is noticeably wider than tall (Fig. 4A), with the lateromedial axis exceeding the dorsoventral axis by more than 10%. Its cranial ornamentation consists of an uneven pattern of ridges, rugosities, and anastomosing grooves as in all teleosaurids. This morphology is distinct from *M*. *laurillardi*, which has a subequal rostrum in height and width (within ±10%) and a pattern of pits and grooves on the dorsal surface of the maxilla. These morphological differences taken together highlight the distinctiveness of *I*. *peninsularensis* when compared to *M*. *laurillardi*, and support its taxonomic separation.

*Indosinosuchus peninsularensis*, *I*. *potamosiamensis*, and the Chinese teleosaurid (IVPP RV 10098) share several distinctive features, including a moderate lateral expansion of the premaxilla, four premaxillary alveoli, and a concave posterior edge of the external nares (Li, 1993; Martin et al., 2019; Johnson, Young & Brusatte, 2020). These characteristics are in contrast to those seen in the holotype of *Peipehsuchus teleorhinus* (IVPP RV 48001), which possesses only three premaxillary alveoli, has lateral premaxillary and maxillary margins almost aligned, and exhibits a convex posterior margin of the external nares (Young, 1948).

The Thai teleosaurid *Indosinosuchus* can be distinguished from the Chinese teleosaurid specimen IVPP RV 10098 based on several cranial characteristics (Figs. 7A–7E). First, the general morphology of the premaxilla differs between the two taxa. Both taxa share the presence of a moderate lateral widening of the premaxilla anterior to the external nares, which is a feature common to the group. However, the degree and shape of this expansion are distinct: in the Chinese specimen (IVPP RV 10098), the premaxilla is markedly expanded laterally, forming a distinct bulbous shape (where the width significantly exceeds the length of the anterior portion), whereas in the Thai material (PRC 205), the premaxilla is more compact and gracile, with the maximum width being only approximately equal to or slightly less than its preserved midline length in the anterior rostrum. Second, the position and relative size of the last premaxillary alveolus are distinct. This alveolus is laterally offset in IVPP RV 10098 and the largest in the series, however in *Indosinosuchus* it is aligned with the third alveolus and has a similar diameter. Third, the premaxillary-maxillary suture in the Chinese specimen displays more pronounced festooning than that observed in the Thai taxon. Given the suite of shared cranial features—particularly the compact premaxilla, the alignment and relative size of the last premaxillary alveolus, the pattern of premaxillary–maxillary festooning, and the external narial opening that is subcircular or slightly ‘8-shaped’ in anterior view—the southern teleosaurid from Ban Nam Pun, Thailand, is best referred to the genus *Indosinosuchus*. These features are better regarded as minor characters coded in the phylogenetic matrix, providing complementary support rather than serving as diagnostic traits *sensu* Martin et al. (2019) and Johnson, Young & Brusatte (2020). Nevertheless, their morphological affinities reinforce the distinction of the Ban Nam Pun material from the Chinese teleosauroid specimen IVPP RV 10098 and argue against their assignment to the same taxon.

**Figure 12:**
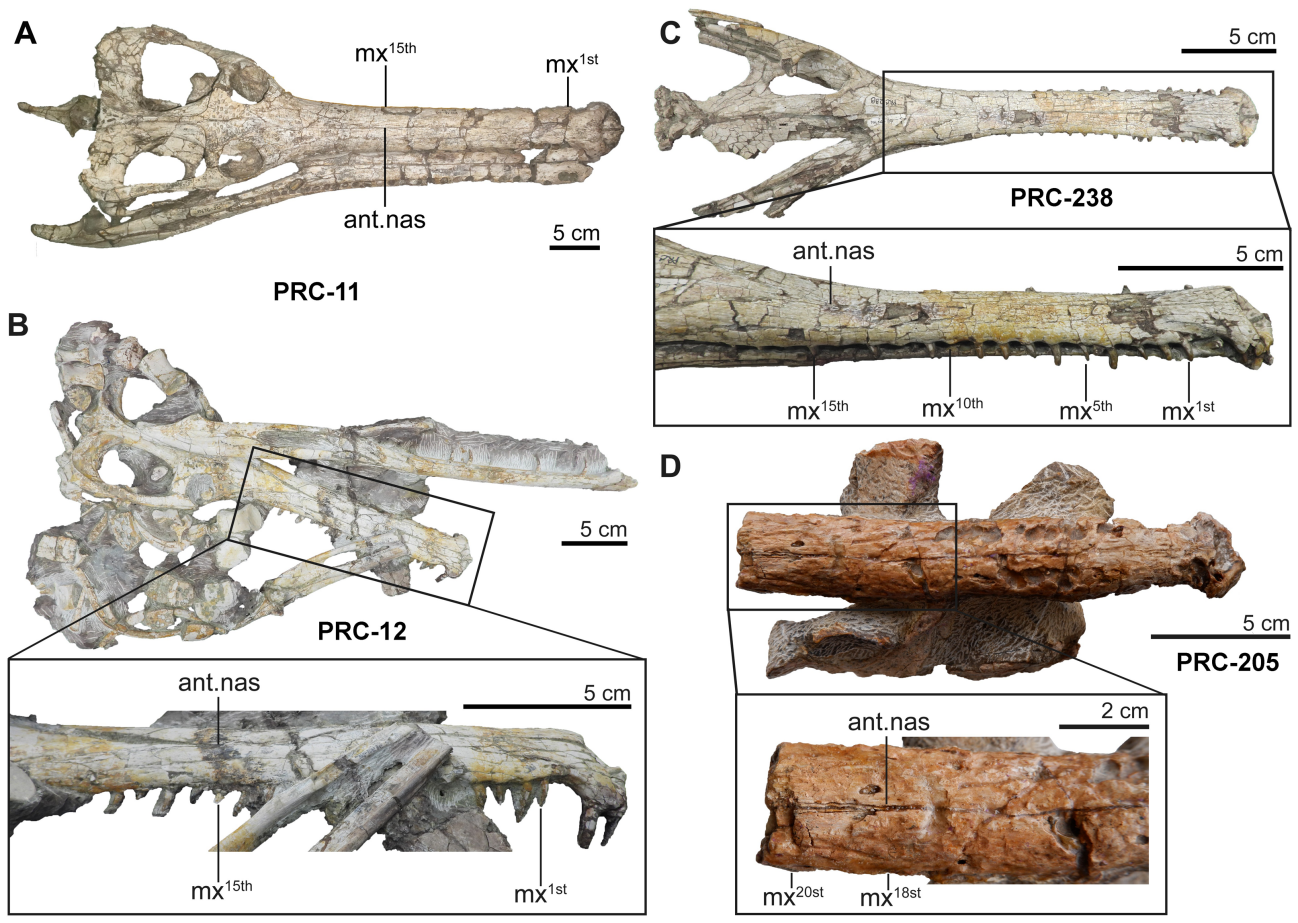
Variation in rostral morphology and anterior nasal position in *Indosinosuchus*: evidence from *I*. *potamosiamensis* and *I*. *peninsularensis* sp. nov. Comparison of rostral morphology in *Indosinosuchus* showing the position of the anteriormost of the nasal (ant. nas) relative to the maxillary alveoli. In *I*. *potamosiamensis* ((A) PRC-11; (B) PRC-12; (C), PRC-238), the anteriormost of the nasal is aligned with the 15th maxillary alveolus. In *I*. *peninsularensis* sp. nov. ((D) PRC-205), the anteriormost of the nasal lies further posteriorly, at the 18th maxillary alveoli. Abbreviations: mx, maxilla.

Accordingly, we propose the establishment of a new species, *Indosinosuchus peninsularensis* sp. nov., based on material recovered from the Middle Jurassic Khlong Min Formation at the Ban Nam Pun locality in southern Thailand. The comparative assessment summarized in Table 2 provides a refined view of morphological variation within *Indosinosuchus*. The dataset incorporates all available cranial specimens of *I. potamosiamensis*, including those formerly referred to *I*. *kalasinensis*, and shows that the variation in this species is relatively limited and consistent across multiple individuals. In contrast, the southern Thai specimens (PRC-205 and 206) display a unique combination of discrete, deformation-independent characters that fall outside the observed range of variation for *I*. *potamosiamensis*. This taxon is morphologically distinct from *I. potamosiamensis*, which is known from the Upper Jurassic–Lower Cretaceous Phu Kradung Formation at the Phu Noi site in northeastern Thailand. The key diagnostic differences, supported by the phylogenetic analysis (Fig. 11), include the following: (1) in *I. peninsularensis*, the anterior tip of the nasals reaches the level of the 18th maxillary alveolus, whereas in *I*. *potamosiamensis* specimens, it extends only to the 14th–15th maxillary alveoli (Fig. 12); (2) the absence of an incisive foramen at the medial contact of the ventral rami in *I*. *peninsularensis* (Fig. 5C) whereas it is present in *I*. *potamosiamensis* (Figs. 5A and 5B), which may reflect a genuine morphological difference; (3) the rostrum of *I*. *peninsularensis* is notably broader than tall (lateromedial axis exceeding dorsoventral by >10%), while that of *I*. *potamosiamensis* exhibits subequal height and width (within ±10%); (4) the maxillary edges of *I*. *peninsularensis* are parallel along both sides up to the tip of the nasal, whereas in *I. potamosiamensis*, the Chinese teleosaurid (IVPP RV 10098), and *Mystriosaurus laurillardi*, the maxillae gradually expand laterally at the level of the nasal tip (Figs. 7 and 8).

**Table 2:** *Indosinosuchus*: diagnostic comparison. Comparison table of diagnostic characters among *Indosinosuchus* species.

**Morphological character**	***I. potamosiamensis*** ** (PRC 11, 12, 238 and 240)**	**Junior synonym of** ***I. potamosiamensis*** ** (PRC 239)**	***I. peninsularensis*** ** sp. nov.** ** (PRC 205–206)**
No. of premaxillary alveoli	4	4	4
Premaxilla shape	Moderately expanded laterally; subequal in length & width	Similar to *I. potamosiamensis* after re-examination	Moderately expanded, subequal length & width, broad but unflared
External nares	Slightly 8-shaped in anterior view	Slightly 8-shaped; “B-shape” taphonomic.	Subcircular to slightly 8-shaped in anterior view (^*^)
Incisive foramen	Present	Present	Absent (^*^)
Anterior tip of nasals	Reaches 14th–15th maxillary alveolus	Reaches 14th–15th maxillary alveolus	Reaches 18th maxillary alveolus (^*^)
Rostrum proportions	Subequal height and width (within ±10%)	Subequal height and width (within ±10%)	Broader than tall (lateromedial axis >dorsoventral by >10%)
Maxillary lateral margins	Gradually expand laterally at nasal tip	Gradually expand laterally at nasal tip	Straight, parallel up to nasal tip (^*^)

**Notes.**

* Autapomorphic characters for *I. peninsularensis* sp. nov.

Although the mid-rostral portion of PRC-205 shows a slight vertical curvature when viewed laterally, the symmetrical configuration of both maxillae in dorsal view (Figs. 7D and 12D) and the consistent thickness of the posterior portion of the maxilla when viewed laterally (Fig. 4A) demonstrate that the rear part of the rostrum largely retains its original geometry. We acknowledge that some degree of deformation did affect the specimen; however, the available evidence suggests that it did not substantially modify the key diagnostic features—particularly the relative breadth and the parallel alignment of the maxillary margins—which are therefore regarded as reliable reflections of the original cranial morphology. Taken together, these differences provide compelling evidence that the Ban Nam Pun material represents a distinct species within the genus *Indosinosuchus*, warranting the formal naming of *I*. *peninsularensis* sp. nov. as the second species of this Southeast Asian teleosauroid lineage.

## Conclusions

The discovery of *Indosinosuchus peninsularensis* sp. nov. from the Khlong Min Formation in southern Thailand provides valuable new insights into the diversity and early evolutionary history of teleosaurids in Southeast Asia. Detailed morphological comparisons (see Table 2) demonstrate that this species is clearly distinguishable from other known Asian taxa, including *I. potamosiamensis* and the Chinese teleosaurid (IVPP RV 10098), based on a unique combination of rostral and osteoderm characteristics. Stratigraphic and paleoenvironmental evidence further supports this distinction, indicating that *I. peninsularensis*, which originated in the Sibumasu Terrane, inhabited shallow marine to peritidal settings during the late Middle to early Late Jurassic. This ecological setting is notably different from the fluvial habitats associated with *I*. *potamosiamensis* from the Indochina Terrane in northeastern Thailand.

A thorough re-evaluation of the holotype and referred material previously assigned to *Indosinosuchus kalasinensis* reveals that the features once regarded as diagnostic are more plausibly explained by taphonomic deformation and intraspecific variation. Morphological traits such as the rostral profile, orbital margins, and the shape of the external mandibular fenestra fall within the range of variation observed in *I*. *potamosiamensis*. Consequently, *I*. *kalasinensis* is here considered a junior synonym of *I*. *potamosiamensis*, refining the taxonomic framework of the genus and supporting a more conservative interpretation of its species-level diversity. Nevertheless, minor inter-population differences merit re-examination in light of future discoveries and more comprehensive material.

In conclusion, current evidence tentatively supports the recognition of only two valid species of *Indosinosuchus* in Southeast Asia—*I*. *potamosiamensis* and *I*. *peninsularensis*. The occurrence of morphologically distinct yet closely related species in stratigraphically and environmentally dissimilar formations emphasizes potential regional endemism and evolutionary divergence. This pattern is consistent with other vertebrate fossil groups from Thailand, such as spinosaurid theropods (Wongko et al., 2019), primitive cryptodiran turtle (Tong, Buffetaut & Suteethorn, 2002), and the hybodontiform shark *Heteroptychodus* (Cuny, Laojumpon & Lauprasert, 2010; Manitkoon et al., 2022), which likewise suggest that geographic separation and environmental variation played key roles in shaping their evolutionary development. This finding not only expands the known temporal and geographic range of the genus *Indosinosuchus*, but also raises important questions regarding the paleobiogeographic history and ecological diversity of teleosaurids in the region. Resolving the evolutionary relationships and paleogeographic patterns of teleosaurids in Southeast Asia will depend on future multidisciplinary studies, including geochronological data, stable isotopic investigations (Martin et al., 2016), and broader comparative frameworks. Although the available material is limited, the diagnostic features presented here support the recognition of a distinct taxon; nevertheless, future discoveries of more complete specimens will be essential to further test and refine the taxonomic interpretations proposed in this study.

## Supplemental Information

10.7717/peerj.20944/supp-1 Supplemental Information 1 Character codings of *Indosinosuchus peninsularensis* sp. nov. in the data matrix of Johnson, Young & Brusatte (2020)

## Institutional Abbreviations

IVPP: Institute of Vertebrate Paleontology and Paleoanthropology (IVPP), Chinese Academy of Sciences, China
PRC: Palaeontological Research and Education Centre, Maha Sarakham University, Maha Sarakham, Thailand
KS: Kalasin Province specimens at Sirindhorn Museum, Sahatsakhan, Thailand;
MSU: Mahasarakham University, Maha Sarakham, Thailand

## Additional Information and Declarations

### Data Availability

The following information was supplied regarding data availability: The CT Scans of PRC-205 and 206 are available at MorphoBank and Figshare: – https://doi.org/10.7934/P5865
– Lauprasert, Komsorn; Martin, Jeremy; Claude, Julien (2026). A new teleosaurid (Crocodylomorpha, Thalattosuchia) from the Sibumasu Terrane of Southeast Asia. figshare. Online resource. https://doi.org/10.6084/m9.figshare.30163993.v2.

### New Species Registration

The following information was supplied regarding the registration of a newly described species: Publication LSID: urn:lsid:zoobank.org:pub:03D87444-73F4-457F-87FF-170B4E92187A Species LSID: Indosinosuchus peninsularensis sp. nov. urn:lsid:zoobank.org:act:85E66457-FBC7-4C52-A0C4-9BFD90ACA4D2
