# Supplementary material for: A new teleosaurid (Crocodylomorpha, Thalattosuchia) from the Sibumasu Terrane of Southeast Asia and a taxonomic reassessment of Indosinosuchus
Source: PeerJ. 2026 Mar 20;14:e20944. doi: 10.7717/peerj.20944 (PMC13007635; doi:10.7717/peerj.20944)
Supplement: Supplemental Information 1 [file peerj-14-20944-s001.docx]

**Supplemental Information:** Character codings of *Indosinosuchus* *peninsularensis* sp. nov. in the data matrix of Johnson et al. (2020).

?????200??0??????0????????01100?0010001010?000-1010001201021-0??????00???0????????????????????????????????????????????????????????????????????????????????????????????????????????0???21??????????????????????????????????????????????????????????????????????????????????????????????????????024?0100?010000000?00?????????????????????????????????????????????????????????????????0???0??????????????????????????????????????????????????????????????????????????????????????????????11?????1??????001?????????-----
